# The Development and Prospects of Socioscientific Issues Teaching in the Context of Immersive Media Technology

**DOI:** 10.3389/fpsyg.2022.877311

**Published:** 2022-04-28

**Authors:** Zeming Kong, Shuya Zhang, Fujin Zhu, Junjun Zhang

**Affiliations:** ^1^College of Humanities and Communications, Hainan University, Haikou, China; ^2^School of Journalism and Information Communication, Huazhong University of Science and Technology, Wuhan, China

**Keywords:** media technology, immersive media, SSI, convergence development, teaching method

## Abstract

Marshall McLuhan once proposed the concept of “global village,” believing that with the help of electronic media, Earth has become indistinguishable from a community, and there is only one Earth for human beings and one world for all countries. Today, with the continuous development of media technology, the concept of a human destiny community has also gained the general consensus of people around the world. The global value of the human destiny community encompasses the interdependent concept of international power, the concept of common interests, the concept of sustainable development, and the concept of global governance. In particular, the concepts of sustainable development and global governance have been advocated by the public, which in turn has led to extensive public discussions on Socioscientific Issues (SSI), in which the teaching of SSI is gradually considered by the international science education community as one of the important goals of science education. The current issues and challenges facing SSI teaching and how immersive media technologies can facilitate SSI teaching have become important issues of keen public interest.

## Introduction

Socioscientific issues (SSI) stems from a reflection on the role of science and technology as a double-edged sword that brings positive utility on the one hand and adverse effects on the other in its application. Therefore, the application of science and technology is always associated with moral and ethical issues, and science and technology also bring about ecological and environmental problems, global governance, etc. These problems are increasingly affecting the survival of human beings, and people will think ethically and morally about the application of science and technology. In the process of thinking, controversial issues are generated, which is the source of SSI (Sadler et al., 2007). SSI is an emerging pedagogical model that goes beyond STS education, which emphasizes the requirements for science education goals but never pursues the ethical issues arising from science and technology. Unlike STS education that simply links science in the form of end results to social applications, SSI education is based on STS education that emphasizes the cultivation of scientific literacy (Bingle and Gaskell, 1994). Furthermore, along with science education, SSI education places more emphasis on introducing social scientific issues into the educational process, allowing students to reflect on the ethical and moral issues that arise in the application of science and technology based on a deep understanding of the nature of science and technology, and allowing students to participate in decision-making on issues with this reflection. By doing so, the educational value of SSI is not only limited to cultivating students’ basic scientific literacy, but also helps improve students’ awareness of ethical and moral issues, while invariably enhancing their reasoning and judgment skills in the process of participating in decision-making. In the context of globalization, the issues brought about by science and technologies extend to all aspects of the world, which poses many new challenges to SSI teaching. At the same time, with continuous development of electronic media technology, the concept of international power, common interests, sustainable development and global governance embedded in the concept of human destiny community provides a new vision for the world to carry out SSI teaching. In response to the global issues brought about by science and technology, it is of great practical significance to study the challenges faced by SSI education in the new environment and new technological conditions, and how to apply immersive media technology to SSI teaching, given the rapid development and widespread application of immersive media technologies.

A large number of existing studies have examined SSI-based learning can enhance students’ disciplinary knowledge and scientific literacy. These articles emphasized interdisciplinarity approach in socio-scientific issues in terms of specific issues. Zowada et al. (2020) states the potential relevance of pesticides for chemistry education in connection with education for sustainable development. Bioscientific advances also put forward numerous new ethical dilemmas. Lombard et al. (2020) advocated cognitive empathy, which is rarely developed in schools, is a crucial skill to address the complex socioscientific issues (SSIs) that recent neuroscience raises. Apart from socio-scientific issues in general, extensive recent studies focus on how SSI-based learning can contribute to science education in the era of the COVID-19. Fooladi (2020) constructed three models as a new composite model that assist communicators and educators understand and analyse complex socio-scientific issues in pandemic. The model is applied on the contradictory issue of Norwegian and Swedish governments’ very different responses to the pandemic (Fooladi, 2020). Reiss (2020) showed how COVID-19 has broadened and deepen the moral philosophy that students typically meet in biology lessons.

Despite the focus on school lessons, some researchers notes the comprehensive abilities for being an outstanding citizen when dealing with socio-scientific issues. These abilities involved many aspects, such as Critical Thinking (CT), Rumor discrimination ability, and other higher-order cognitive thinking skill for making decisions. For instance, Barzilai and Chinn (2020) proposed diverse perspectives on how post-truth problems related to scientific and socio-scientific issues might be educationally addressed. Evagorou et al. (2020) conduct a qualitative study of a classroom of 10–12-year-old students working collaboratively in argumentation and modeling and further explore how primary school students use their models whilst arguing about a socio-scientific issue. Likewise, Ke et al. (2021) proposed the significance of learning and using multiple models for students in the context of SSI.

Some researches revealed relevant individual attitudes toward SSIs. Ke et al. (2020) focus on students’ perceptions of SSI-based learning and uses semi-structured interviews from 33 students in a midwestern U.S. high school to investigate students’ perceptions of SSI-based learning and how to support students in considering the epistemic aspects of SSI learning. While Nida et al. (2020) collected questionnaires from 99 Indonesian science teachers and explored their experience and perceptions toward SSI-based education. The authors found that almost all of the participants saw potential in SSI-based education for the character formation of students, but most of the respondents did not implement SSI-based teaching very often in their lessons because of a range of limitations (Nida et al., 2020).

Numerous scholars have conducted extensive research in diverse teaching methods used in SSI-based Education. Suryani et al. (2020) conducted a Classroom Action Research in order to find out how implementing Group Investigation (GI) learning model combined with SSI improve students’ Problem-solving skills. Some authors have driven the further development of online SSI-based education. Rodrigues Martins et al. (2020) introduced a curricular approach where a research group consisted of schoolteachers, undergraduate and graduate students analyzed controversies and fake news in the production of a web page. Similarly, Puig Mauriz et al. (2021) carried out an empirical study with a group of secondary students engaged in diverse online activities that required them to practice critical thinking and argumentation for dealing with coronavirus information and disinformation, and aimed to examine students’ critical assessment about SSIs.

There are also a small number of studies have involved immersive media technology in SSI education. Filter et al. (2020) found that immersive technology could stimulate interest about nature related to the socio-scientific issue, even among people who did not already hold positive attitudes toward that issue. The authors pointed out that immersive technology provide nature experiences with positive affective learning outcomes (Filter et al., 2020). However, the study focused on nature experiences in VR and was not an educational experience.

Overall, there has been very little discussion about SSIs in an Immersive Media Technology Context. On the one hand, the existing studies are predominantly focused on a single topic of SSIs, and lacked a systematic classification of these topics. On the other hand, the role of Immersive Media technologies and its effects in SSIs teaching were not discussed in-depth. This manuscript establishes an evaluation system for SSIs education, systematically and quantitatively examines the factors that influence SSIs education, and discusses the role of Immersive Media in public understanding of SSI in a bid to address the gap.

## Construction of Socioscientific Issues Teaching Measurement Indicators

Back in the 1920s, when radio technology was first invented, McLuhan used the term “global village” to describe the great impact of radio technology on human society. McLuhan used the term “global village” as such, “But one thing is for sure: the discovery of electromagnetic waves has reshaped the synchronous ‘field’ of all human things, so that the human family exists in the ‘global village’ state” (McLuhan, 2014), or something like “it is a new world of global village” (McLuhan, 2011). Nowadays, with the rapid development and high prosperity of information technology and electronic media, the term “global village” is widely known and has become a buzzword used on many occasions. Now people usually use “global village” to describe Earth has been no different from the community. People in any country or region can rely on electronic media technology to be linked together as one. Human beings live in a common Earth and a world, electronic media technology has brought everyone closer. The concept of “global village” and the human destiny community both describe the state of Earth becoming increasingly integrated today; moreover, the concept of human destiny community has a rich connotation. At present, among the results of domestic academic research on SSI, there are fewer literatures that analyze the impact of the concept of human destiny community on SSI from the perspective of globalization and human destiny community, which also makes the research from this particular perspective novel and innovative. We can see that each of the four aspects embedded in the global value of the human destiny community provides a multidimensional and new perspective for SSI research.

### International Power Concept and Socioscientific Issues

For many centuries, the struggle for international power among different countries and groups has been dominated by wars, which have brought untold disasters to mankind and caused many tragedies in human history. Today, with the increasingly frequent transnational movement of science and technology, capital, population and other elements, the world is tightly linked into a whole. A bond of interest is formed between countries in order to realize their respective interests. Therefore, a country that wants to assert its international power and thus achieve its interests does not necessarily have to go to war, as it did before. The economic interdependence between countries has contributed to the slowing down of the international situation. During the formation of the international system, the contrast between the level of science and technology between countries can be used as a measure of the international position occupied by a country. Therefore, all countries hope to enhance their economic strength and international status through innovative research in basic science and the application of new technologies, so as to take their place in the international system. It is for this reason that the moral and ethical issues of countries in applying science and technology to gain international status have been raised, and these issues have become a hot topic of public concern, which has given rise to SSI on the international concept of power. The common topics of SSI on the international view of power and its manifestations include the following: (1) Discussion of “hegemonism” in science and technology. Scientific and technological “hegemonism” manifests itself in the form of international suppression, bullying, and isolation of countries with a later start, a weaker foundation, and a lower level of science and technology by some countries that have started the scientific and technological revolution earlier and are therefore stronger in science and technology. It also manifests itself in unilateral protectionism in science and technology, imposing sanctions on technology companies of other countries. (2) Discussion of nuclear war. The emergence of nuclear weapons is a great progress in the history of human science and technology, but at the same time, it will also cause a crisis of human’s own survival. Once a nuclear war breaks out, mankind will face an unprecedented disaster. The famous scientist Albert Einstein once said candidly that mankind should destroy nuclear weapons to avoid possible nuclear war. Currently, however, mankind obviously has no intention of destroying nuclear weapons, which has to arouse people’s concern and discussion on this issue. (3) The discussion of artificial intelligence technology. AI technology is the frontier and focus of world science and technology competition at present and in the near future, so it is also a hot area of science and technology innovation in various countries. However, will AI benefit mankind? Stephen Hawking has questioned it, and he warned that people should be wary of AI. The discussion of AI technology also appears in people’s daily lives.

### View of Common Interests and Socioscientific Issues

The view of national interest has different connotations in different times. During the monarchy period, the interests of the state represented only the interests of the monarch and the royal aristocracy in the country. In the 20th century, the interests of the international community were described as a zero-sum relationship with an exclusive nature. Under the conditions of economic globalization, the concept of national interests has been defined in a new way. First of all, there is a deeper reflection on the traditional view of national interests. Today, as the world is increasingly integrated into a “global village,” one country may serve its own interests and realize its own interests to the detriment of others, because the interests of all countries have become highly intertwined and formed a chain of interests that binds all countries together. Globalization has broken the limitation of time and space, which makes the people of the world more and more closely connected to each other and their interests more and more intertwined. However, conflicts of interests and disputes among countries still exist, and the phenomenon of unreasonable and unjust distribution of international interests still exists (Xinxin, 2019). In this context, the issues that arise include the following: (1) the discussion of international “unilateralism.” At present, there are still big countries uphold the zero-sum game thinking, in order to maintain their own interests at the expense of other countries’ interests. Without regard for the wishes of the majority of countries and people, they individually or take the lead in withdrawing from or challenging the rules and systems that have been established or agreed upon to maintain international, regional, and collective peace, development, and progress. They also manifest behaviors and tendencies that have destructive effects and consequences on global or local peace, development, and progress. (2) For the discussion of world food safety issues. When food safety problems occur in one country, this country may export food with safety problems to other countries in the world, thus jeopardizing the food safety interests of other countries. (3) A discussion of refugee movement. Nowadays the increasing development of transportation has made it possible to move waves of refugees. This puts a country in a dilemma, where it may be in its interest to keep refugees out of the country, but it will be subject to humanitarian condemnation.

### Sustainable Development Concept and Socioscientific Issues

Among the concepts of the human destiny community, the concept of sustainable development is the most discussed topic in daily life. The concept of sustainable development that conforms to the development interests of all countries in the world has gradually become the broadest consensus around the world. This is because in the context of economic globalization, environmental issues have evolved into a human issue, which are related to the survival of human beings. As we all know, the natural environment is the natural basis for the existence and development of human society, and it is impossible for human beings to survive alone without a certain natural environment. If the momentum of further deterioration of the earth’s environment is not reversed, the civilization created by human society may suffer from extinction and go down the drain. Therefore, how to achieve harmony between man and nature and how to achieve sustainable development in this harmonious relationship is a major issue facing humanity. The contradictory state between man and nature originated from the technological revolution, and the transitional development of industry caused unprecedented damage to the natural ecological environment. Starting from the first technological revolution, which took place at the end of the 18th century, mankind applied science and technology to the production process on a large scale. From the use of the steam engine, which symbolized the beginning of the first technological revolution, to the arrival of the internal combustion engine on the industrial stage in the second technological revolution, to the application of more modern and advanced science and technology in the production process today, the conflict between man and nature has become more and more acute. Against this backdrop, “*The Rio Declaration on Environment and Development*,” “*The Johannesburg Plan of Implementation,”* and “*The Future We Want*” and other achievement documents with the core concept of sustainable development have emerged. The concept of sustainable development is increasingly becoming a consensus issue for countries around the world to consider when formulating their development plans. Led by the value of sustainable development, SSI focuses on reflecting on the environmental problems brought by science and technology to human beings. At present, the environmental problems plaguing human beings include global warming, the sharp decline of biodiversity, the deterioration of air quality, and the shortage and deterioration of water resources, etc. The SSI on sustainable development is also centered on these issues. (1) Discussion of global warming. Although the international community has made a collective effort, and the “*Paris Agreement”* established in 2015 set the goal of keeping global warming within 2°C of pre-industrial levels for nearly a century and striving to achieve the target of 1.5°C (United Nations, 2017). However, the trend of global warming has not been reversed, and the environmental problems brought about by global warming have prompted people to reflect on the nature of science and technology. (2) Discussion on the deterioration of water resources. Improper discharge of industrial wastewater and domestic wastewater can directly cause water quality deterioration and affect the safety of aquatic organisms. If a country discharges too much wastewater into the sea then the wastewater may affect other countries with the flow of ocean currents. People gradually have a new understanding of science and technology and its application in the discussion of these issues. Some foreign scholars believe that we still need to use science and technology to change nature, making it meet people’s needs. In addition, technology is once again considered as a means that can promote the development and health of ecosystems, both individual organisms and human communities. Therefore members of these communities enhance the health and life of these ecosystems in their actions (Gunderson et al., 2010, pp. 167–195). Guided by the concept of sustainable development, SSI provides a guide for human beings to use advanced science and technology to solve environmental problems and achieve harmonious relationship between human and nature by reflecting on the environmental problems brought about by science and technology. This is the reason why SSI on the concept of sustainable development can be so extensively discussed.

### Global Governance and Socioscientific Issues

The scientific concept of global governance was introduced in the report “*The World is Our Neighborhood*” of the Commission on Global Governance (CGG) published on the 50th anniversary of the United Nations. The core concept of global governance is that, in the context of the era of global integration, the international action subjects are diversified. The subjects to deal with global issues also consist of different governments, intergovernmental organizations, non-governmental organizations, multinational corporations, etc. It is only through the interaction of these pluralistic subjects in joint participation and mutual consultation that the current global issues can be solved in a good and practical way. The global governance issues facing human society are diverse, ranging from development issues, security issues, to environmental and other multifaceted issues. How to formulate a reasonable global governance norm in the global context and make it universally mechanism and morally binding to the global governance subjects has become the key to properly deal with these global issues. Throughout the context of global integration, various global problems caused by science and technology are not well solved because of the absence of such a well-regulated global governance system. Therefore, the SSI on the concept of global governance revolves around these issues. (1) Discussion of the ethical norms of biotechnology. Biotechnology is also a globally developed technology field, and biotechnology without appropriate ethical norms to restrict its use may lead to global biosafety issues and moral and ethical problems. The development of cloning technology, for example, has led people to think about the ethical issues involved. In particular, nowadays the emergence of cloning technologies such as gene cloning has forced people to be wary of such biotechnology. Another example is the question of whether genetically modified foods are harmful to human health, which makes people fearful when they talk about genetic modification. Furthermore, the problem of microbial leakage caused by experiments on viruses and other microorganisms has a direct impact on the survival of human beings. (2) Discussions by regional organizations. An international organization formed by some countries to promote the unity and solidarity of the region to which they belong and to strengthen mutual cooperation in politics, economics, diplomacy, defense and security. For example, the European Union, BRICs, G8 Group, etc. The issue of how to achieve benefit sharing has been widely discussed.

## Model Construction

**FIGURE 1:**
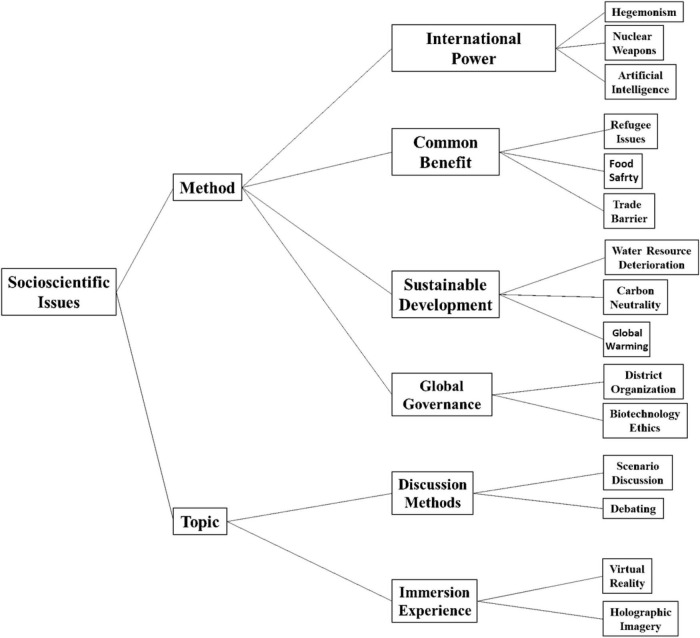
Hierarchy of socioscientific issues (SSI) educational assessment.

In order to establish an assessment system for SSI education and to systematically and quantitatively examine the influence of various factors on SSI education, this study uses a hierarchical structure model for specific model construction. The model consists of two primary criteria, four secondary criteria and 15 tertiary criteria, which are expressed as shown in Figure 1.

In the multi-level structure model, the importance of each indicator is bound to vary, and it is necessary to quantitatively describe the same level elements by comparing them two by two through the 9-level scale method, construct a judgment matrix, and calculate the weights occupied by each criterion to assist in decision analysis.

### Delphi Method of Collecting Expert Opinions

To enhance the rationality and refinement of the model, this study was based on the Delphi method, and the importance of the above factors was judged by distributing questionnaires to 20 experts. The expert group selected for this study was composed of teachers from several universities and Ph.D. students with education-related backgrounds with remarkable representativeness, all with higher education and certain teaching experience, to ensure the authenticity, scientific validity, and accuracy of the feedback data. In order to eliminate interference, the experts evaluated the questionnaires based solely on their personal perceptions during the process of distribution, without meeting, communicating or interfering with each other.

### Hierarchical Analysis to Determine the Weight of Subjective Indicators

Hierarchical analysis was first proposed in the 1970s by American operations researcher T. L. Saaty, a professor at the University of Pittsburgh, as a method of analysis for multiple indicator problems (Saaty and Kearns, 1985). It operates by combining expert opinion and relevant literature to divide the influencing factors of a problem into an ordered hierarchy of interrelated factors and to determine the weights of the criteria at each level.

Based on Thomas Satie’s literature, this study used a 5-level scale approach, in which experts were asked to subjectively assign weights to the indicators in the model. The hierarchical analysis method is divided into five scales: equally important, slightly important, quite important, extremely important, and absolutely important, with the values 1, 3, 5, 7, and 9, in addition to four scales between these five scales, namely 2, 4, 6, and 8. The assessment scales and their interpretations are shown in Table 1.

**TABLE 1:** Assessment scales and their interpretations.

Assessment scales	Interpretations
1	Both guidelines are equally important
3	The former is slightly more important than the latter
5	The former is quite more important than the latter
7	The former is extremely more important than the latter
9	The former is absolutely more important than the latter
2, 4, 6, 8	Intermediate value of adjacent scales
Inverse of the above scale	The inverse comparison of two criteria, such as criterion i to criterion j with a scale of xij and vice versa with 1/xij

The judgment matrix Q is obtained after pairwise comparison of each indicator at the same level of hierarchy as follows. In Q, α*_*i*_*, α*_*j*_* (*i*, *j* = 1, 2,…, *n*) denote factors. α*_*ij*_* denotes the relative importance value of α*_*i*_* to α*_*j*_*, satisfying α*_*ij*_* > 0 and α*_*ij*_* = 1/α*_*ij*_*, and the judgment matrix is expressed as follows:


$$Q=\left[\begin{array}{cccc} a_{11} & a_{12} & \ldots & a_{1\,n}\\ a_{21} & a_{22} & \ldots & a_{2\,n}\\ \vdots & \vdots & \vdots & \vdots \\ a_{n\,1} & a_{n\,2} & \ldots & a_{n\,n} \end{array}\right]$$

In order to avoid contradictions in the logic brought about by subjective decisions during pairwise comparison of the criteria of each level of SSI education by the relevant experts, a consistency test is needed to detect the reasonableness of the judgments made by the expert questionnaire. The test method is as follows: CR = CI/RI, where CI = (λ_max_ − *n*)/(*n* − 1).

In the above equation, CR is the consistency ratio, CI is the consistency index, λ_max_ denotes the maximum eigenvalue of the matrix, and RI is the random consistency index, whose values are shown in Table 2.

**TABLE 2:** Randomized indicators.

n	1	2	3	4	5	6	7	8	9
RI	0	0	0.58	0.90	1.12	1.24	1.32	1.41	1.45

According to Satie’s suggestion, when CR < 0.1, the degree of consistency of the judgment matrix is more desirable; otherwise, the values of indicators in each layer need to be readjusted. In the judgment matrix of this study, all the CR values are less than 0.1, so the expert decision data pass the consistency test. After calculation, the weight data in Table 3 can reflect the influence of each factor on SSI education in a more comprehensive way. Among the primary criteria, the quality of information is the most important. Among the secondary criteria, timeliness of feedback service, update rate of the system and ease of information comprehension are the most important.

**TABLE 3:** Weights table of education evaluation indicators with socioscientific issues (SSI).

Criteria name	Global weights	Peer weights
Topic selection	0.4916	0.4916
Teaching style	0.5084	0.5084
International power	0.1121	0.2281
Common interests	0.1236	0.2514
Sustainability	0.1354	0.2754
Global governance	0.1205	0.2451
Discussion style	0.2463	0.4845
Immersion	0.2621	0.5155
Hegemonism	0.0357	0.3181
Nuclear weapons	0.0372	0.3317
Artificial intelligence	0.0393	0.3502
Unilateralism	0.0376	0.304
Food safety	0.0432	0.3495
Refugee issues	0.0428	0.3466
Carbon neutrality	0.044	0.3252
Global warming	0.0467	0.3452
Water resources deterioration	0.0446	0.3296
Regional organizations	0.062	0.5143
Biotechnology ethics code	0.0585	0.4857
Scenario discussion style	0.1218	0.4945
Debate style	0.1245	0.5055
Virtual reality (online)	0.1349	0.5149
Holographic imagery (offline)	0.1271	0.4851

## Technology Immersion: The Integration of Immersive Media Technology and Socioscientific Issues Teaching

Immersive media refers to the use of computer generated technologies such as VR, AR that isolate the users from the real world while presenting reality as a three-dimensional virtual environment (Herrera et al., 2018). The fundamental idea of immersive media is to allow the participant to see the world from the first-person perspective, which could lead to greater audience involvement (De la Peña et al., 2010). Immersive Media is an emerging technology that is being increasingly used in teaching and learning as it continues to develop and mature. In our model of the SSI teaching assessment system, instructional methods and content are included in the same level of criteria, and the results of the expert opinion collection and calculation show that instructional methods are given more weight than content. This indicates that the choice of a teaching method is very critical to the effectiveness of SSI teaching. Virtual worlds, serious games, simulations, and augmented reality are enabling students and instructors to connect with content and each other in novel ways. Many have noted the new support these media provide and their impact on important pedagogical constructs such as presence, immediacy, and immersion (Bronack, 2011). Because of the many characteristics of immersive media technologies themselves, such as the ability to break the limits of time and space and to construct virtual realities and thus immerse people, their application to science teaching can have unexpected effects. In the process of science teaching, the biggest advantage of immersive media technology is to provide students with a three-dimensional experience, thus it has the effect of greatly enhancing the enthusiasm of students to learn actively. For example, in high school physics classes, immersive media technology can bring students to the vast universe of stars, so that they can personally experience the magic and wonders of the universe. In SSI teaching, immersive media technology can likewise gain a wide scope of application. However, the integration of SSI teaching with immersive media technologies still faces many difficulties, and the results of the model show that the traditional SSI instructional approach of debate style still has a higher weighting than that of immersive instruction. Today, with rapid development and increasing popularity of immersive media technologies, combining SSI teaching with immersive media technologies has become a new expectation for SSI education.

### Teaching Socioscientific Issues Online

In today’s rapid development of information technology, the Internet has entered thousands of households. Especially, the popularity of cell phones has increased people’s access to the Internet and reduced the cost. According to the data, the Internet penetration rate in China has reached 94.6% by 2019. Thus, it becomes possible to implement SSI teaching online. When we compare the offline viewing SSI teaching with the online immersion SSI teaching, we know that the online immersion SSI teaching has more weight. This is because online immersive SSI teaching is not only convenient, but also maximizes the characteristics and advantages of immersive media technologies that are not limited by time and space. Although there are many weaknesses in the current online teaching resource platforms, such as the lack of sharing among different teaching resource platforms, slow updating of teaching resources, and teachers’ inability to understand students’ online learning, etc. (Kone et al., 2000). However, it is undeniable that online immersive SSI teaching has a wide application prospect. Teachers can build SSI scenarios online for students to conduct online SSI learning and discussion, which can fully engage students in SSI discussions compared with traditional classroom teaching of only 50 mins. Moreover, with the addition of immersive media technology, the SSI scenarios built online are more impactful for students, allowing them to face the conflict situations online through the virtual and three-dimensional experiences brought by immersive media technology. By exposing students to the intricacies of issue conflict, they can experience how conflict arises in the process of value formation, conflict, and decision making, and guide them to actively consider how to apply due process to resolve conflict so that conflict can become a driving force for democratic development has become an important experience in teaching SSI abroad (Yucheng, 2013). Inspired by this experience, building a shared, comprehensive and open online teaching platform for SSI and making full use of the advantages of immersive media technology in SSI teaching have become the main trend for conducting SSI teaching at present and in the future.

### Establishing a Multi-Functional Immersive Teaching Experience to Promote Socioscientific Issues Teaching in a Comprehensive Manner

The development of immersive media technology has broken the limitations of traditional SSI teaching methods. Traditional SSI teaching methods are limited to book teaching, oral teaching, electronic audio-visual teaching, etc. These traditional SSI teaching methods are increasingly inadequate to meet the demands of SSI teaching in the context of globalization and the increasing global issues today. People are no longer satisfied with the traditional means of accessing SSI resources through reading books, especially for children who are full of the unknown and eager to learn about the world. Traditional SSI teaching methods are not only limited in satisfying children’s curiosity, but also not motivating children to think actively. Immersive media technology certainly provides a new opportunity to revolutionize SSI teaching and learning. Immersive media technologies can provide high quality, intuitive and multi-sensory stimulating SSI situations to the educated population. Multi-functional immersive SSI teaching and learning experience centers with low entry barriers can be established in various places, and science teachers can regularly lead children to visit, experience and learn in the experience centers, so that they can realistically and genuinely feel the multifaceted problems brought by technology to human beings and drive them to think actively. In the comparison of the secondary guidelines, the view of sustainable development has the greatest weight, because environmental protection and sustainable development are becoming major issues of concern. The global warming issue, the deterioration of water resources, and the carbon neutrality issue, which are related to the view of sustainable development, are all given considerable weight in the three-level guidelines we developed. The peer weights they occupied are 0.3452, 0.3296, and 0.3252, respectively. Therefore, we can conduct immersive SSI teaching for these issues of sustainable development. For example, we can use immersive media technology to build an experience hall that can virtually simulate the ecological and environmental crises of the Earth, and in the process of teaching biology courses, we can lead students to visit this experience hall to make them feel the ecological and environmental crises that Earth is suffering from. This will lead them to think about the science nature and establish a correct view of the science nature. In addition, although the weight of nuclear weapons in the tertiary guidelines is not significant, we are always facing the threat of nuclear war and nuclear leakage. With the development of immersive media technology, we can even establish experience museums to simulate nuclear war and nuclear leakage and other situations, which also become the future of immersive SSI instruction.

### Simultaneous Immersive Media Technology Training and Socioscientific Issues Instructional Training for Science Teachers

Compared to traditional SSI teaching, there is a certain threshold of technical proficiency in using immersive media technologies for SSI teaching. For most science teachers, the ability to use and manipulate immersive media technology to build highly feasible SSI instructional contexts is limited by the science teachers’ proficiency with this technology. Therefore, training science teachers on immersive media technology becomes critical to the success and effectiveness of immersive media technology in facilitating SSI teaching. In addition, training science teachers in immersive media technology should be accompanied by training them in SSI teaching, and this is an even more important aspect. First, training should be conducted to strengthen science teachers’ understanding of SSI content knowledge. Research studies by Liu Enshan’s team as well as by Tidemand and Nielsen (2016) from abroad have shown that the current understanding of science teachers about SSI content knowledge is not ideal and there are misconceptions about SSI content knowledge (Bing and Enshan, 2021). This predisposes teachers to neglect the cognitive and ethical development of students in SSI teaching. Therefore, enhancing the training of science teachers’ SSI content knowledge in practice becomes a prerequisite for SSI teaching using immersive media technologies. Second, science teachers should be trained to teach SSI on an individual basis. The different subject matter knowledge taught by science teachers in different disciplines has resulted in variability in SSI teaching in different disciplines, and the training of science teachers in SSI teaching should also be conscious of this variability. For instance, teachers of biological sciences should be made aware of the current problems in the world due to the use of biological sciences, so that they can properly teach SSI in biological sciences classrooms in conjunction with immersive media technologies that are appropriate for the biological disciplines. Along with training science teachers in immersive media technologies, training them in SSI teaching can create a highly qualified science teacher force that will continue to develop a feasible space for the integration of immersive media technologies in SSI teaching.

### Application of Immersive Media Technology for Socioscientific Issues Teaching Should Target Global Issues

Under the leadership of the value of human destiny community, SSI teaching must focus on global issues, including development issues, governance issues, political and economic conflicts, sustainable development issues, and other issues faced by human beings. This has become an irreversible trend in SSI teaching. In the secondary guidelines, the peer weights of international power perspective, common interest perspective, sustainable development perspective and global governance perspective are 0.2281, 0.2514, 0.2754, and 0.2451, respectively. To carry out immersive SSI teaching accordingly, scientific and reasonable teaching plans and contents should be formulated according to the different weights. In the process of continuously promoting the integration of immersive media technology and SSI teaching, more attention should be paid to the characteristics and advantages of immersive media technology, i.e., the ability to build SSI teaching contexts by constructing virtual scenarios. For example, in SSI teaching about sustainable development, the use of immersive media technology to build virtual scenarios of environmental deterioration not only allows students to intuitively experience the environmental problems in their own countries, but also allows them to see the impact of the environmental deterioration problems brought by technology to Earth on the oceans, glaciers, climate, and various other aspects. This is beneficial for students to expand their perspective and understand the nature of science. The question of how immersive media technologies can be integrated into SSI teaching, and how to focus on global issues in this integration, is a critical topic for research.

## Conclusion

This manuscript has explored the multidimensional perspectives provided by the international power perspective, common interest perspective, sustainable development perspective, and global governance perspective on SSI under the value of human destiny community. At the same time, an assessment system of SSI teaching is constructed in order to quantitatively examine the impact of each factor on SSI teaching. By collecting experts’ opinions through Delphi method to measure the indicators of the model, we found that it is more critical to choose a type of SSI teaching method to enhance the effectiveness of SSI teaching than the choice of SSI teaching content. In the context of accelerated globalization, there are a variety of issues raised by science and technology, and there are also a variety of SSIs generated. SSI teaching methods must keep pace with them, which requires us to continuously improve teaching methods and adopt different teaching methods for different SSIs. Immersive SSI teaching is better than traditional debate-based SSI teaching both in terms of weighting and teaching effectiveness. Therefore, this manuscript proposes four feasible suggestions on how to achieve the integration of immersive media technology and SSI teaching. Combined with the results of the model, this manuscript suggests that we should actively explore the application of immersive media technology in SSI on international power, common interests, and global governance, and build practical SSI teaching contexts. The corresponding SSI teaching methods can be based on offline discussion-based teaching and online immersion-based teaching. For SSI teaching on sustainability, the use of immersive media technology will be more widespread. We can establish a multi-functional immersive media technology experience hall and build a variety of virtual situations for SSI teaching, so that students can personally feel the changes that are taking place in the global environment. Thus students can actively reflect on the global warming problem and find ways to solve it, and they can therefore care for and protect nature more. In addition, it is worth mentioning that regional organizations in the global governance perspective are also attracting more attention. This is because in the context of the accelerated evolution of globalization, a variety of regional organizations are increasingly influencing people’s lives and have become an indispensable part of global governance. Therefore, teaching in this area should be appropriately increased in the content of immersive SSI. The application of immersive media technology in SSI teaching is still very promising, and how to apply it to SSI teaching and its effect is still a new problem for us. This manuscript tries to provide theoretical guidance on how to achieve the integration of immersive media technology and SSI teaching in the context of globalization.

## Data Availability Statement

The raw data supporting the conclusions of this article will be made available by the authors, without undue reservation.

## Ethics Statement

Ethical review and approval was not required for the study on human participants in accordance with the local legislation and institutional requirements. Written informed consent for participation was not required for this study in accordance with the national legislation and the institutional requirements.

## Author Contributions

JZ: conceptualization, methodology, and software. ZK: data curation, writing – original draft preparation. SZ: visualization and investigation. FZ: software and validation. All authors contributed to the article and approved the submitted version.

## Conflict of Interest

The authors declare that the research was conducted in the absence of any commercial or financial relationships that could be construed as a potential conflict of interest.

## Publisher’s Note

All claims expressed in this article are solely those of the authors and do not necessarily represent those of their affiliated organizations, or those of the publisher, the editors and the reviewers. Any product that may be evaluated in this article, or claim that may be made by its manufacturer, is not guaranteed or endorsed by the publisher.
